# The identification of XPR1 as a voltage- and phosphate-activated phosphate-permeable ion channel

**DOI:** 10.21203/rs.3.rs-4457423/v1

**Published:** 2024-12-11

**Authors:** Hongjiang Wu, Liang Sun, Tong Huo, Theodore G. Wensel, Frank T. Horrigan, Zhao Wang

**Affiliations:** 1 Verna and Marrs McLean Department of Biochemistry and Molecular Pharmacology, Baylor College of Medicine, Houston, TX 77030, USA; 2 Department of Integrative Physiology, Baylor College of Medicine, Houston, TX 77030, USA; 3 These authors contributed equally.; 4 Department of Molecular and Cellular Biology, Baylor College of Medicine, Houston, TX 77030, USA; 5 CryoEM Core (Advanced Technology Core), Baylor College of Medicine, Houston, TX 77030, USA; 6 Department of Materials Science and NanoEngineering, Rice University, Houston, TX 77030, USA; 7 Department of Molecular and Cellular Oncology, Division of Basic Science, The University of Texas MD Anderson Cancer Center, Houston, TX 77030, USA; 8 Lead contact

## Abstract

Maintaining a balance of inorganic phosphate (Pi) is vital for cellular functionality due to Pi’s essential role in numerous biological processes. Proper phosphate levels are managed through Pi import and export, facilitated by specific Pi transport proteins. Although the mechanisms of Pi import have been extensively studied, the processes governing Pi export remain less understood. Xenotropic and Polytropic retrovirus Receptor 1 (XPR1) has been identified as the only known Pi export protein in mammals, playing a key role in facilitating Pi efflux from cells. Malfunctions in XPR1 are associated with human diseases, such as primary familial brain calcification and certain cancers, highlighting its critical role in maintaining Pi homeostasis. In this study, we introduce the cryogenic electron microscopy structure of human XPR1 (hXPR1), unveiling a structural arrangement distinct from that of any known ion transporter, with a topology not identified in previous computational predictions. Our structural results suggest that hXPR1 may operate as an ion channel, a hypothesis supported by patch clamp recordings revealing hXPR1’s voltage- and Pi-dependent activity and large unitary conductance. Using proteoliposomal uptake assays, we demonstrate that purified and reconstituted hXPR1 catalyzes transport of Pi. Further analysis, including the structure of hXPR1 in presence of Pi, and functional effects of mutating a putative Pi binding site, leads us to propose a plausible ion permeation pathway. Together, our results provide novel perspectives on the Pi transport mechanism of XPR1 and its homologues.

## Introduction

Xenotropic and Polytropic retrovirus Receptor 1 (XPR1), also known as SLC53a1 of the solute carrier (SLC) superfamily, is a multi-pass membrane protein initially identified in mice as the cell surface entrance receptor for murine xenotropic and polytropic retroviruses^1,2^. The function of XPR1 was later found to mediate inorganic phosphate (Pi) export from the cytosol to extracellular space^3–5^. The protein is well conserved phylogenetically across all eukaryotes, and the Pi-exporting activity has been demonstrated in various orthologues^6–10^. Given the role of Pi in many key cellular processes including energy production, biosynthesis, and cell signaling, its intracellular concentration is tightly regulated, in part through controlling Pi import and export^11,12^. XPR1 is the only known inorganic phosphate exporter in mammals, is present in most cell types^12^, and thus plays a central role in maintaining cellular Pi homeostasis. XPR1 mutations have been associated in patients with primary familial brain calcification (PFBC)^4,5,13–17^, a genetic neurodegenerative disorder marked by progressive bilateral calcification distributed primarily in the basal ganglia region^18^. In addition, the upregulation of XPR1 has been implicated in several cancers, facilitating cancer proliferation, migration, and invasion^19–25^. Considering the critical role of XPR1 in regulating Pi homeostasis and the current knowledge gaps in our understanding of its connection to XPR1-related diseases, a systematic study to explore its structural-functional mechanisms is of great importance.

All XPR1 homologues are composed of two major functional domains: the N-terminal cytosolic SPX (SYG1/PHO81/XPR1) domain, and the transmembrane domain (TMD). The SPX domain was discovered as an intracellular phosphate sensor^26^, and the XPR1-mediated Pi export activity is regulated by SPX binding to inositol polyphosphates^27–29^. Secondary structure predictions proposed that the XPR1 TMD is composed of 8 transmembrane α-helices^4^. Part of the TM region belongs to the EXS (ERD1/XPR1/SYG1) domain family, which was demonstrated to be essential for proper localization to the plasma membrane and Pi export activity for plant orthologue PHO1^30^. Whereas crystal structures have been reported for the SPX domain^26^, a detailed structure-function relationship study of the full-length XPR1 protein could enhance the understanding of the transport mechanism of XPR1 and its critical role in regulating phosphate homeostasis.

## Results

### Cryo-EM structure of hXPR1

We expressed full-length human XPR1 (hXPR1) in HEK293S GnTI^−^ cells, purified the protein in detergent mixture (Supplementary Fig. 1) and determined the structure by cryogenic electron microscopy single particle analysis (cryo-EM SPA) in the absence of any Pi or known ligands. The overall resolution of the apo-hXPR1 (ligand-free) map reached to 3.4 Å with the transmembrane region extended to 2.7 Å. The quality of the map was sufficient to allow accurate assignments of backbones and side chains within the TMD (Supplementary Figs. 2 and 4a). The cytosolic domain of apo-hXPR1 was relatively poorly resolved compared to the TMD, possibly due to flexibility, but we were able to perform rigid body docking and flexible fitting to accommodate the previously determined SPX crystal structure^26^ (PDB:5IJH) into the density map. The identification of the position of the SPX domain enables the unambiguous assignment of TMD topology.

**Fig. 1:**
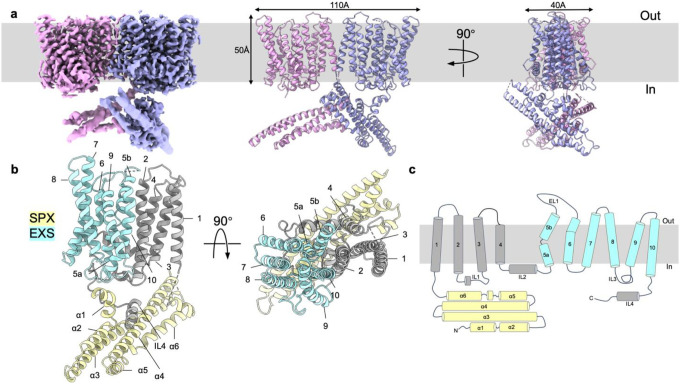
Overall structure of apo-hXPR1. **a.** Cryo-EM density map (left) and cartoon representations of the atomic model (middle and right) of apo-hXPR1 dimer viewed in the membrane plane from two orthogonal directions. Two protomers are colored magenta and lavender. The densities of cytosolic domain and TMD are displayed at a contour level of 8.17σ and 5.04σ respectively. The grey box in the background indicates the membrane bilayer. **b.** Cartoon representations of an hXPR1 monomer viewed from the side and from top-down. The SPX domain is colored in yellow, EXS domain in light blue, and the rest of the protein in gray. **c.** Topology of a monomeric hXPR1.

hXPR1 in detergent mixture forms a homodimer. The overall TM domain has a trapezoidal shape with dimensions of 110 × 40 × 50 Å (Fig. 1a and Supplementary Movie 1). For each protomer, the cytosolic SPX domain connects to the TMD *via* an unresolved flexible linker, and the C-terminus is situated in the cytosol as well (Fig. 1b). The TM domain of each protomer consists of 10 transmembrane α-helices as opposed to 8 helices hypothesized previously (Fig. 1bc). The dimeric interaction is mediated predominantly within the TM region by TM1, and the dimer interface has a buried surface area of 449 Å^2^. It is interesting to note that the previously predicted EXS domain, which was hypothesized to contain a long cytoplasmic loop and three TM helices, spans the TM5 to TM10 segments with the predicted loop amounting to TM6 and TM7 (Fig. 1b). There are 4 long intracellular loops connecting TM2 to TM3, TM4 to TM5, and TM8 to TM9, and the C-terminal region is also intracellular. The only substantial extracellular loop connects TM5 to TM6 (Fig. 1c).

It was previously reported that XPR1 adopts a unique fold compared to other members of SLC family.^31^ To determine if the helix arrangement of TM domain belongs to any other known structural fold, which might potentially provide insight into the Pi transport mechanism, we used the structure similarity search engine DALI^32^ to compare the TM domain structure of hXPR1 to known proteins in the Protein Data Bank. Strikingly, the result indicated that the hXPR1 is not similar to any known Pi transporters or any other secondary transporters with “alternating-access” mechanisms in general.^33^ This structural distinction from ion transporters suggests hXPR1 could potentially mediate Pi permeation *via* an uncommon mechanism. The closest resemblance of hXPR1 is to the archaeal ion-translocating rhodopsin family, where the topological arrangement of TM5–10 from hXPR1 matches loosely to TM2–7 from a light-driven chloride ion-pumping rhodopsin^34^(Supplementary Fig. 5). Such structural similarity suggests that TM5–10 might carve out an isolated space from the membrane lipid environment that creates a pathway for ion permeation, as seen in ion-translocating rhodopsin with its TM2–7. In addition, the absence of any blockade (*e.g*. retinal) within this isolated space may allow a continuous path which could potentially facilitate passive diffusion as seen with ion channels.

### hXPR1 exhibits voltage- and Pi-dependent ion channel activity, and Pi transport activity

**Fig. 2:**
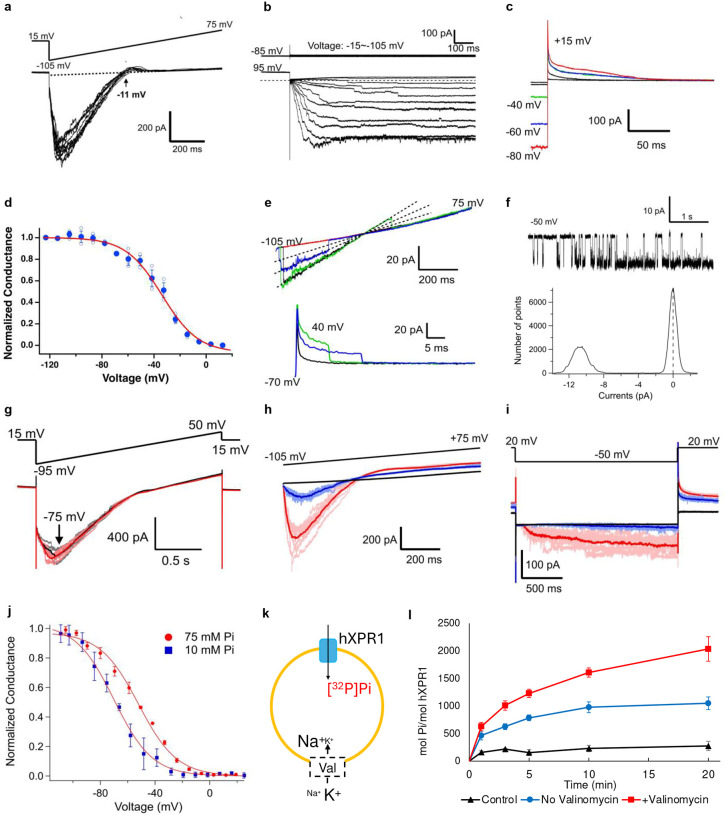
XPR1 exhibits voltage- and Pi-dependent ion channel activity and Pi transport. **a.** Inwardly rectifying macroscopic currents evoked from GUVs with purified hXPR1 by repeated voltage ramps (−105 mV to +75 mV) from a holding potential of +15 mV **b.** Slowly activating inward currents are evoked by hyperpolarizing voltage-pulses following a prepulse to +95 mV in GUVs with hXPR1, but not in detergent control. **c.** Outward tail current decay at +15 mV following hyperpolarizing voltage pulses as in **b. d.** Mean normalized G-V relations for hXPR1 from tail currents (mean ± SEM, n=3 replicates from one GUV) show the voltage-dependence of steady-state activation, which is fit by a Boltzmann function (z = −1.8 ± 0.2 *e*, V_1/2_= −34 ± 2 mV, mean ± SD). **e.** Unitary current activity during voltage ramps or +40 mV pulses evoked from and inside-out patch from a HEK293S GnTI^−^ cell expressing hXPR1. a-e were recorded in inside-out patches with external 0 Pi NMDG-MSA and internal 20 Pi, 0.1 Ca, K-MSA solutions **f.** Unitary XPR1 current recorded from HEK293 cell with single open level and associated all points histogram at −50 mV with external 1 Pi NMDG-citrate and internal 20 Pi, 0.1 Ca, K-MSA solutions. **g.** XPR1 currents evoked from a GUV patch by voltage ramps with Pi as the sole internal anion (black) or with 10 Cl- (red) are superimposable, indicating the channel is permeable to Pi and Cl- at this concentration makes little contribution. XPR1 currents evoked from a GUV patch by voltage ramps (**h**) or −50 mV pulses (**i**) are enhanced as internal Pi is increased from 10 mM (blue) to 75 mM (red), and almost undetectable in 0 Pi (black) using 10 Cl K-MSA internal solutions with external 0 Pi NMDG-MSA. Thick curves represent an average of 10 traces. **j.** Mean steady-state G-V relations in 10 and 75 mM Pi show that increased internal Pi shifts activation to more positive voltages by 18 mV. G-Vs at both [Pi] were normalized to the maximal conductance measured in 75 mM Pi. **k.** Schematics of the [^32^P] Pi transport assay with proteoliposomes. A membrane voltage difference was generated using potassium gradient and valinomycin, making the voltage at the external side of the liposomes more negative with respect to the lumen. **k, l.** Time-dependent accumulation of 0.1mCi/mL [^32^P] Pi with 25mM non-radioactive carrier Pi in hXPR1-containing proteoliposomes without the addition of valinomycin (circles with blue lines), with valinomycin (squares with red lines), and in empty liposomes with the addition of valinomycin (triangles with black lines) as control. Means and s.e.m. of time-dependent uptake plotted (n=6)

To further investigate the ion-channel hypothesis, patch clamp electrophysiology experiments were conducted using giant unilamellar vesicles (GUVs) reconstituted with purified hXPR1. Currents recorded from excised inside-out patches in response to voltage-ramps exhibited a strongly rectifying behavior with large 0.5 nA inward currents at voltages near −100 mV but little or no current at positive voltages (Fig. 2a). Inward currents evoked by 1 s pulses to different voltages following a prepulse to +95 mV activate to a steady state, with faster activation at more negative voltages (Fig. 2b, lower panel). Small steps and stochastic fluctuations in current are evident, suggestive of ion channel activity. Importantly, control experiments lacking hXPR1, but with the same concentration of detergent, exhibited no appreciable current over the same voltage range and ionic condition (e.g. Fig. 2b, upper panel). Transient outward (tail) hXPR1 currents could be evoked by stepping to +15 mV following activation of inward current at negative voltages (Fig. 2c). The voltage-dependence of steady state activation (Fig. 2d) was determined by plotting the mean normalized conductance-voltage relation (G-V), measured from the tail current amplitude following 1 s pulses to different voltages, and is fit by a Boltzmann function with apparent charge of −1.8 *e* and half-activation voltage of −34 mV. A similar voltage-dependence of macroscopic current was observed in whole cell recording of HEK293S cells transfected with hXPR1 (Supplementary Fig.6ab). This voltage-dependence implies that the tail current decay at +15 mV reflects deactivation (i.e. channel closure) and that the rectifying behavior observed with voltage ramps is due to voltage-dependent channel gating as opposed to a strong dependence of open channel conductance on voltage. The latter is evident from hXPR1 currents recorded from excised patches from XPR1-expressing HEK293S cells during voltage ramps (Fig. 2e, upper panel), which reproduce the rectifying behavior in GUVs (Fig. 2a) but with an order of magnitude smaller current and stochastic activity consistent with a small number of channels. Dashed lines indicate three open levels of equal conductance whereas the red trace with no current fluctuations represents a sweep where channels remained closed. Stochastic closing events can also be observed following steps to +40 mV (Fig. 2e, lower panel), and a different patch shows steady-state activity at −50 mV with the corresponding all-points histogram (Fig. 2f). The linear relation between current and voltage indicates that the conductance of the open channel is voltage-independent with a unitary conductance of 134 pS, based on the difference in slopes of the dashed lines in Fig. 2e.

The negative reversal potential of XPR1 current (Figs. 2a and e, arrows) indicates that the channel is not selective for Pi, as the intracellular and extracellular solutions contained 20 mM and 0 Pi respectively. However, this observation does not rule out the possibility that the channel conducts Pi together with other ions. Indeed, large inward currents were recorded with 75 mM Pi as the sole internal anion (Fig. 2g), supporting that XPR1 is permeable to Pi. In addition, increasing internal Pi from 10 to 75 mM greatly increased peak current during voltage ramps or −50 mV pulses (Fig. 2hi) without altering unitary current amplitude at −50 mV (Supplementary Fig.6cde). This indicates that XPR1 channel activity is Pi-dependent. The enhanced activity of XPR1 in 75 mM Pi is due to a shift in the steady-state G-V relation to more positive voltages relative to 10 mM Pi without change in maximal conductance (Fig. 2j), as well as a speeding of activation kinetics (Supplementary Fig. 6c). Small macroscopic XPR1 currents could also be recorded in 0 Pi (Supplementary Fig. 6c) but only at voltages more negative than −70 mV suggesting a further difference in the voltage-dependence of activation between 0 and 10 mM Pi. Unitary current fluctuations at −75 mV in 0 Pi (Supplementary Fig. 6f) were comparable in magnitude to those observed in 10 or 75 mM Pi at −50 mV, implying that the small macroscopic XPR1 current in 0 Pi (Supplementary Fig. 6c) reflects a failure to maximally activate the channel at the most negative voltages tested. XPR1 does not appear to be selective for Pi versus methanesulfonate. The currents recorded in 0 Pi, with methanesulfonate as the primary internal anion indicates that the channel is permeable to this ion. Furthermore, the change from 75 mM to 10 mM Pi, which involved substitution of 100 mM methanesulfonate for Pi, had no appreciable effect on the unitary current amplitude (Supplementary Fig. 6de). The internal solution for this experiment also included 10 mM Cl^−^. However, switching from 0 to 10 mM Cl^−^ in the presence of 75 mM Pi had no effect on mean current amplitude following XPR1 activation (arrow Fig. 2g) implying that Cl^−^ at this low concentration makes little or no contribution to XPR1 conductance. The selectivity of the channel was not investigated in detail owing in part to the strong dependence of channel activity on internal [Pi]. Currents in Fig. 2 were recorded with extracellular solutions containing NMDG as the main cation and methanesulfonate (Figs. 2a–e) or citrate (Fig. 2f) as the main anion and low Cl^−^, to reduce the number of potential permeant ions and to minimize conductance through native channels in HEK293 cells.

One advantage of the strong dependence of XPR1 activity on internal [Pi] is that in GUV recordings only channels oriented with their cytoplasmic side facing the vesicle lumen should be activated under typical inside-out recording conditions where the luminal side is exposed to high Pi. To test this hypothesis and confirm that XPR1 in GUVs are reconstituted in both orientations, we recorded from inside-out patches with intracellular (20 mM Pi) solution in the pipette and external (NMDG-methanesulfonate) solution in the bath. Under these conditions, outward XPR1 currents were recorded at positive voltages exhibiting rectification consistent with channels oriented with cytoplasmic side out. (Supplementary Fig. 6g). That our GUV data in Figure 2 reproduces results from HEK cells therefore can be accounted for by the fact that we only applied high Pi on the luminal side.

To test whether the isolated protein is functional for Pi transport, we conducted proteoliposome flux assays and found that under the same buffer condition with citrate in which currents were observed by patch clamp recordings in Fig. 2f, liposomes reconstituted with hXPR1 protein, but not empty liposomes, showed time-dependent accumulation of [^32^P] Pi. This transport was enhanced when the membrane potential was perturbed using a potassium gradient and the potassium ionophore valinomycin (Fig. 2kl). These results could be accounted for by voltage increasing the driving force or open probability, consistent with the electrophysiological experiments. That is, since the external side of the vesicles was exposed to high (25 mM) Pi, only XPR1 with the cytoplasmic side facing out should have been activated, and the imposed voltage (negative on the external side) should favor increased channel activity as well as increased driving force for Pi entry.

### The putative ion-permeation pathway

**Fig. 3:**
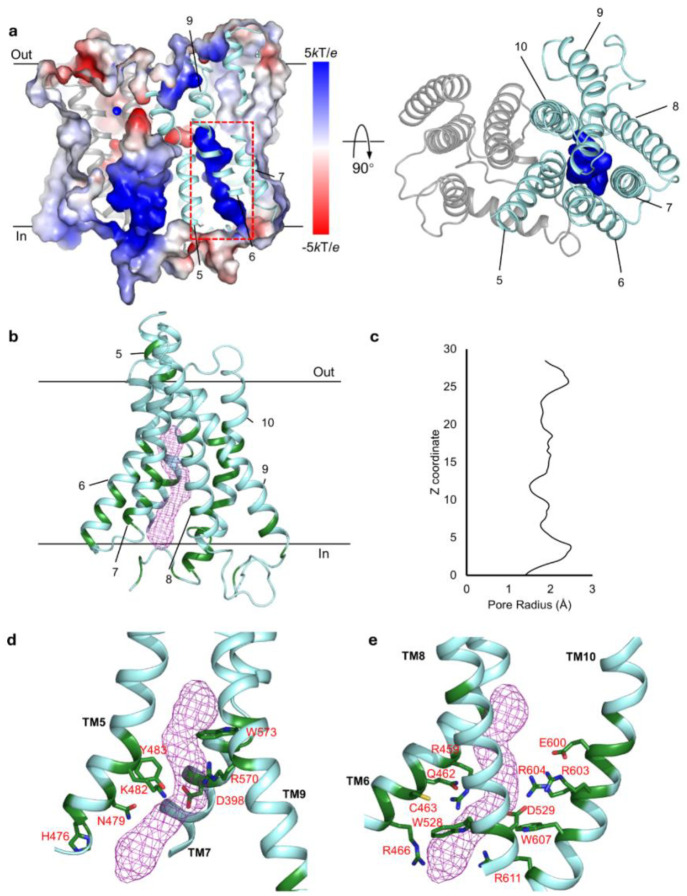
The putative pore of hXPR1. **a.** (left) The solvent-accessible surface of the hXPR1 TMD colored by ±5 *k*T/*e* electrostatic potential calculated using APBS^58^. The secondary structures of TM1–4 are shown in grey and TM5–10 in light cyan. The positively charged vestibule formed at the center of the barrel-shaped helical bundle of TM5–10 is boxed in red. Two solid black lines indicate the membrane boundary. (right) The electrostatic surface–potential map depicting the same vestibule alone, view orthogonally from the extracellular space. **b.** The putative pore location in hXPR1 inside the 6-helix barrel colored light cyan, and pore pathway is depicted as purple mesh. The residues on the TM5–10 that are conserved among hXPR1, atPHO1, and scSYG1 are colored in dark green. **c.** Pore radius along the z coordinate. **d, e** Detailed view of the green-colored conserved pore-lining residues shown in stick model on TM5, 7, and 9 **d**, and TM6, 8, and 10 **e**.

The ion-channel like conductance of hXPR1 observed by patch clamp recording elicited a closer examination of the TM domain of hXPR1 to identify potential ion permeation pathways. We found that each of the transmembrane segments TM1–4 is surrounded by the detergent environment individually and thus relatively isolated, suggesting that these four helices might not participate in ion translocation across the membrane. On the other hand, the TM5–10 are organized sequentially into a 6-helix bundle in a clockwise arrangement (viewed from the cytoplasmic side), forming a barrel-shaped structure. Aside from TM9, all helices within the barrel are oriented roughly perpendicular to the membrane surface. TM9 on the other hand is tilted to ~45° with respect to the membrane. The protein’s electrostatic surface reveals a highly positively charged vestibule at the center of the 6-helix bundle (Fig. 3a). This tunnel-like pathway is open to the cytoplasmic side and extends to the center of the protein. The positive surface of this vestibule arises from a series of positively charged residues including Arg459, Arg466, Lys482, Arg570, Arg603, Arg604, and Arg611, and this overall positivity of the cavity is consistent with a pore that can conduct anions. To visualize the putative ion permeation pathway, we used the CAVER program^35^. The identified pore generally overlaps with the positive vestibule. The pore is accessible to solvent on the cytosolic side but is closed to the extracellular side in the apo-hXPR1 structure (Fig. 3b). The first õne-third of the pore leading from the cytosolic entrance is formed by TM5a, 6, 7, 8, and 10. The tilted helix, TM9, meets the others in the middle, and all TM5–9 contribute to the central portion of the pore. The portion leading to the extracellular exit is closed by insertion of TM9 into the 6-helix barrel (Fig. 3b). The overall diameter of the pore is around 4 Å, with a narrowest restriction of 3 Å. (Fig. 3c). Many of the surface-lining residues within this putative pore are conserved across different species among hXPR1, plant PHO1, and yeast SYG1 (Fig. 3de and Supplementary Fig. 7), suggesting this passage may be conserved among XPR1 homologues.

### Structure of hXPR1 in presence of Pi

**Fig. 4:**
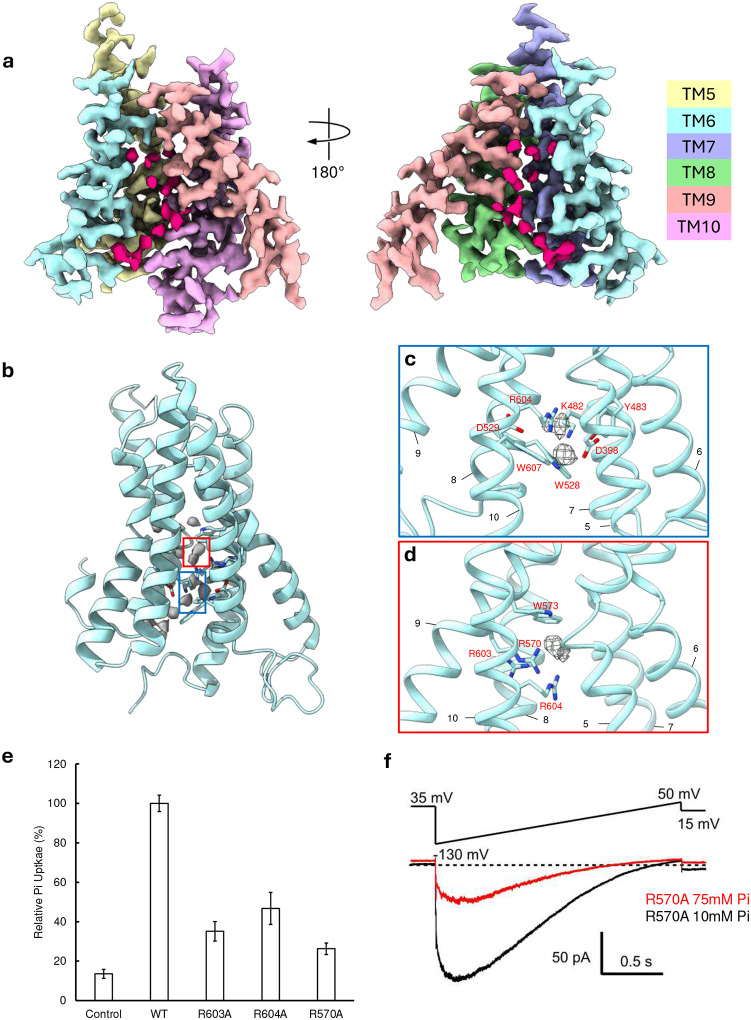
Putative ion coordination sites. **a.** The density map of TM5–10 of Pi/InsP_6_-hXPR1, with each TM helix colored individually at a contour level of 10.96σ. The non-protein isolated densities within the pore are colored in pink red. Densities from TM5 and TM10 (right), or TM7 and TM8 (left) are removed to expose the pore. **b.** The string of putative ion densities in grey depicted at 10.96σ contour level with the cartoon representation of Pi/InsP_6_-hXPR1 TM5–10 structure. Densities corresponding to the two putative ion coordination sites are box in red and blue. **c.d.** Close-up views of the two putative ion coordination sites indicated in the colored boxes in **b**, with the ion density shown at 5.35σ contour level**. e.** Relative Pi transport of the alanine mutations of three arginine residues within the red-colored putative Pi binding in **c.** The relative transport was measured at the 20-minute time point with membrane valinomycin. (n=4). **f**. XPR1 R570A currents evoked from a GUV patch by voltage ramps are decreased as internal Pi is increased from 10 mM (black) to 75 mM (red), using 10 Cl K-MSA internal solutions with external 1 Pi, 10 NMDG-Cl.

To identify potential phosphate binding sites we solved the structure of hXPR1 in buffer containing 25 mM sodium phosphate and 1 mM phytic acid (InsP_6_), as inositol polyphosphates are known to facilitate Pi export upon binding to the SPX domain^28^. The soluble SPX domains of Pi/InsP_6_-hXPR1 map were poorly resolved compared to the TMD, as evident from the 2D classification analysis (Supplementary Fig. 3b), 3D reconstructions with either C1 or C2 symmetry imposed did not yield a structured and resolvable soluble domain. Thus, C2 symmetry was imposed for the final reconstruction of Pi/InsP_6_-hXPR1 map. The resolution of the resulting density map reached 2.3 Å, which is sufficient to recognize ions in the density (Supplementary Fig. 3, 4b and Supplementary Movie 1). The overall TMD structure of Pi/InsP_6_-hXPR1 does not differ significantly from that of apo-hXPR1, with an RMSD of only 0.271Å. However, in the Pi/InsP_6_-hXPR1 map, we identified a string of isolated, non-protein densities within the putative pore surrounded by TM5–10, that were not observed in the apo-hXPR1 map (Fig. 4a). It is highly likely that these densities represent locations for Pi ions as they travel through the pore. Based on these densities, we identified two locations along the putative pore which could serve as Pi coordination sites (Fig. 4b). The first site is situated near the narrowest restriction of the pore, where two positive charged residues Lys482 and Arg604 sandwich the putative Pi density, with sidechains of other surrounding conserved residues Asp398, Tyr483, and Asp533 located more distally (Fig. 4c). The second site is near the extracellular end of the putative pore, in which the putative ion density is surrounded by three positive residues Arg604, which also participates in the first putative coordination site, in addition to Arg603 and Arg570 (Fig. 4d). These three positively charged core residues form a sequential arrangement with two consecutively on one helix and the other on an adjacent helix. Interestingly, this type of core interaction pattern is similar to the phosphate recognition region in triose-phosphate/phosphate translocator of plant, which also has three positively charged residues Lys204, Lys362 and Arg363 organized into a similar pattern (Supplementary Fig. 8)^36^. As such, these core residues may form a key Pi coordination site in the XPR1 putative pore. Located above the three arginine residues is Trp573, the aromatic residue whose sidechain is positioned perpendicular to the pore. Although the string of densities extends beyond Trp573 (Fig 4b), these extended densities are surrounded by non-conserved neutral residues.

Mutations of each of the three arginine residues (Arg570, Arg603, and Arg604) in the second putative Pi coordination site to alanine significantly impaired the Pi uptake in the flux assay (Fig. 4e). In addition, in patch clamp assays, while large currents could be recorded from R570A in 10 mM Pi with methanesulfonate as the main anion, currents were greatly reduced in 75 mM Pi (Fig. 4f), an effect opposite to that observed with WT XPR1 (Fig. 2g). This suggests the mutation selectively reduces Pi permeability, consistent with a role of R570 in Pi coordination.

The positions of residues lining the surface of the putative pore have little difference between the apo-hXPR1 and Pi/InsP_6_-hXPR1 maps, as evident from an TM5–10 RMSD of 0.267 Å between two structures. Thus, the dimension of this pore in the Pi/InsP_6_-hXPR1 structure is very similar to that of apo-hXPR1, with the narrowest diameter of 3 Å. In addition, in both structures the TM9 forms a single continuous transmembrane helix, with the top segment inserted directly into the pore, effectively blocking the exit towards the extracellular space. Thus, we propose both structures represent the closed state of hXPR1.

### The C-terminal tail bridges SPX domain and TMD

In the cytosolic helical bundle of one of the protomers of the apo-hXPR1, we identified a short α-helix that does not map to the SPX domain. The density map of this protomer displays a well-resolved connection between this short cytoplasmic helix and the end of TM10, the last TM helix of TMD (Supplementary Fig. 9a). This connection allows us to build a portion of this protomer’s C-terminal cytoplasmic tail. This short helix, which we denote as intracellular loop 4 (IL4), was assigned to residues 636 to 646 (Supplementary Fig. 9b) linked directly to TM10 via a loop. Given the different orientations of SPX domains with respect to the TMD between two protomers in the apo structure (Supplementary Fig. 9c) and the unresolvability of SPX in the Pi/InsP_6_ structure, we hypothesize that SPX domain is flexible and might undergo conformational changes in response to different conditions. The cytoplasmic tail, with one end connecting directly to TMD and the other forming a short helix that bundles with the SPX domain, potentially serves to bridge between the SPX domain and TMD and provides the architectural basis for the allosteric regulation of SPX domain on TMD.

## Discussion

In this study, we investigated the structure-function relationship of human XPR1. Our structures revealed that hXPR1 is dissimilar to known transporters but has features consistent with ion channel function: TM5–10 form a helical barrel, and within this barrel a central cavity is identified which reveals a partial pathway with appropriate diameter and charge to conduct anions; the additional densities seen coordinated to positively charged sidechains within that pathway in the presence of Pi likely represent Pi coordination sites.

Electrophysiological recordings from hXPR1 in excised patches revealed large unitary currents with a linear open channel I-V relation in HEK293 cells, and large macroscopic inward currents in GUVs, including in the absence of Pi or with Pi as the sole internal anion, all supporting the conclusion that XPR1 can function as an ion channel that is permeable to Pi and relatively non-selective for anions. The Pi transport activity was further confirmed using proteoliposomal flux assays. The lack of structural similarity between XPR1 and known transporters, together with the identification of channel-like structural topology including a pore architecture with putative Pi binding sites, supports that XPR1 transports Pi as a channel rather than as a Pi transporter with uncoupled ion channel activity, a hypothesis further supported by observations that mutations of the key arginine residues within one of the putative Pi coordination sites impaired Pi uptake in the flux assay, and one of them showed reduced Pi permeability in patch clamp recordings.

The rate of Pi transport (~10 Pi per XPR1 s^−1^, from the 1 min time point) is orders of magnitude less than the charge movement through the open channel measured with patch clamp at −50 mV (Fig. 2f) owing to several factors that cannot all be quantified. First, in the flux assay, the initial rate is likely to be underestimated owing to the time resolution of the measurement. Second, the membrane voltage is not controlled and is likely to favor a low Po (<0.1) if V is near 0 based on the V-dependence of activation (Fig. 2d). Third, the fraction of XPR1 protein molecules that are functional in the flux assay and have correct membrane orientation to be activated by high external Pi is unknown. Finally, in the patch clamp assay, Pi flux represents only a fraction of the total charge movement as the channel is not selective for Pi over the predominant anion methanesulfonate.

Strong inward rectification and large unitary conductance clearly distinguished XPR1 activity from native channels occasionally observed in HEK293 cells. The inward rectification arises from voltage-dependent activation of the channel at negative voltages. Activation is also Pi-dependent, with little activity in the absence of Pi and shifts in the G-V relation to more positive voltages as [Pi]_i_ is increased. The channel appears to attain a high open probability, near unity, at maximally effective voltages in high [Pi]_i_, as unitary currents activated at −100 mV in 20 mM Pi during voltage ramps exhibit no sign of transient closure (Fig. 2e), and the maximal macroscopic conductance at 10 or 75 mM Pi is constant (Fig. 2i).

The mechanistic basis of voltage-dependent activity is unknown but is unlikely to simply reflect voltage-dependent block of the pore by impermeant ions, given the slow activation kinetics that required up to 0.5 s to reach equilibrium (Fig. 2b). Alternative possibilities include voltage-dependent conformational changes in the protein (*i.e*., a voltage-sensor domain), or a dependence of channel opening or closing on voltage-dependent binding of a permeant ion to a site or sites within the pore, a mechanism which has been proposed by various groups to contribute to the voltage-dependent activation of CLC_0_ chloride channels^37^. That activation is shifted to more positive voltages as internal [Pi] is increased is consistent with the notion that Pi binding to a site in the pore may contribute to the voltage-dependence of activation.

With our structures we could map the locations of PFBC mutations (Supplementary Fig. 11a). Many of the mutations were known to locate on the SPX domain, which could potentially disrupt the SPX regulation of the Pi export activity. On the other hand, our structures provide novel perspectives on how mutations on other parts of hXPR1 could lead to diseases. Three mutations are located within the TM5–10 helical barrel forming the putative ion-permeation pore: Arg459, Arg570, and Ile575 (Supplementary Fig. 11b). Specifically, mutations of Arg459 and Arg570 have been shown to lead to reduced Pi export without affecting the protein expression levels^5,15^. These two arginine residues are conserved (Supplementary Fig. 7), with Arg459 located near the narrowest constraint and Arg570 within the putative Pi coordination site (Fig. 3e and Fig. 4c), in support of the hypothesis that Pi permeates through the putative pore. Moreover, both our flux assay and electrophysiological recordings showed that mutation of R570A impaired Pi transport, which supports its role in the putative Pi coordination site and may help explain the pathological mechanism of the PFBC-causing variant R570L.

In addition, three disease-associated mutation sites, Asn619, Arg624, and Ile629, are located within the C-terminal cytoplasmic tail on the loop connecting TM10 to IL4 (Supplementary Fig. 9b). Asn619 and Arg624 are also conserved across XPR1 homologues (Supplementary Fig. 7), and these mutations were documented to reduce XPR1-mediated Pi efflux as well^5^. Combined with the potential flexibility of the SPX domain, these results suggest a novel role for the C-terminal cytoplasmic tail in bridging the SPX domain with the TMD to achieve allosteric regulation.

Both our structures likely represent a closed state based on the pore size and the TM9 blockade towards the extracellular side. It is possible that an alternative, perhaps transient, state not observed in our data, allows Pi exit to the extracellular side. It is still unclear how the pore would open. To explore reasonable alternative structures, we used Alphafold2 to predict structures for the transmembrane domain^38^. When compared, the helix arrangements in our experimental structures and the most probable prediction are quite similar, with one major difference focusing on TM9. AlphaFold2 predicts that TM9 is broken into two segments with a kink in the middle, and the segment closer to the extracellular space is rotated away from the 6-helix bundle (Supplementary Fig. 9a). In this conformation, TM9 no longer blocks the ion permeation pathway, and the pore is open to both sides of the membrane (Supplementary Fig. 9b). Trp573 resides next to the kink, and in our closed structure the side chain of Trp573 is situated directly above the putative Pi-binding site, with Arg570 being one helical turn away (Supplementary Fig. 9a). Thus, we propose the hypothesis that a bent conformation of TM9 at Trp573 may open hXPR1 to allow Pi efflux. Trp573 resides next to the kink, and in our closed structure the side chain of Trp573 is situated directly above the putative Pi-binding site, with Arg570 being one helical turn away (Supplementary Fig. 9a). In Pi transporter SLC20, a kink is observed in a helix lining the Pi binding pocket at a conserved tryptophan residue, and the helix-bending mechanism was proposed to control the opening and closing of the gate that allows the Pi release^39^. Thus, we propose the hypothesis that a bent conformation of TM9 at Trp573 may open hXPR1 to allow Pi efflux.

In summary, our structural and functional data established that hXPR1 transports Pi as an ion channel whose activity is regulated by intracellular Pi concentration and membrane voltage. It is likely that if XPR1 functions as a non-selective anion channel in cells, its activity must be tightly regulated. The requirement that XPR1 activates only at negative voltages with high intracellular Pi assures that the channel will only be open under conditions where the electrochemical gradient favors Pi efflux. In addition, the Pi export activity of XPR1 in cells is thought to be critically dependent upon the presence of higher order intracellular inositol pyrophosphates such as InsP7 and InsP8^27–29^, which are only transiently generated as a result of excess Pi conditions^40–42^. Additional means of regulating XPR1 activity have also been reported.^10,43,44^ These regulatory mechanisms may allow the channel to act as an “escape valve” for Pi that is only transiently activated, and this pattern of Pi efflux could potentially be linked to the phenomena of rapid Pi release documented in various cell types^45,46^, specifically in pancreatic β-cells in which XPR1 was established to mediate the “phosphate flush”^47^. Our results provide insights into XPR1’s role in maintaining intracellular Pi homeostasis and reveal the structural and functional impacts of mutations causing PFBC, enabling further investigations into their mechanisms and approaches to therapeutics.

## Methods

### Expression and Purification of hXPR1

The cDNA of human XPR1 (Uniprot: Q9UBH6) was synthesized with a Strep-tag II peptide fused at the C-terminus, and cloned into pBacMam vector for expression in HEK293S GnTI^−^ cells^48^.

The purification was carried out at 4 °C. ^−^ The cell pellet from 2L of HEK293S GnTI culture was resuspended in 100 mL lysis buffer containing 20 mM Tris, 150 mM NaCl, and 2 mM MgCl_2_ buffered at pH 7.4, supplemented with 1 protease inhibitor cocktail tablet (Roche) and 5 μL of nuclease (Thermo Fisher) per 50 mL buffer. The cells were directly solubilized by adding 1.5% (w/v) n-dodecyl-β-d-maltoside (DDM, Anatrace) and 0.15% (w/v) cholesteryl hemisuccinate (CHS, Anatrace) for two hours and were centrifuged at 180,000 × g for 1 hour. The supernatant containing detergent-solubilized hXPR1 protein was loaded onto StrepTactin HP affinity purification column (Cytiva) and washed with wash buffer containing 20 mM Tris pH 7.4, 150 mM NaCl, 0.005% (w/v) glyco-diosgenin (GDN, Anatrace), 0.005% (w/v) lauryl maltose neopentyl glycol (LMNG, Anatrace), and 0.0001% CHS. hXPR1 protein was then eluted with wash buffer supplemented with 5 mM desthiobiotin (Sigma Aldrich). The eluted protein was concentrated using centrifugal filter unit with 50 kDa cut-off down (Milipore) to 500 μL volume. For apo-hXPR1 structural studies, the concentrated protein was loaded onto Superose 6 10/300 GL size-exclusion column (Cytiva) pre-equilibrated with wash buffer. For hXPR1 in presence of Pi/InsP6, the size-exclusion column was pre-equilibrated using 25 mM sodium phosphate, 150 mM NaCl, and 1 mM phytic acid (InsP_6_) at pH 7.4 with the same GDN/LMNG/CHS detergent mixture concentration.

### Size Exclusion Chromatography-Multi-Angle Light Scattering (SEC-MALS)

Data were collected using a Dawn Ambient light scattering instrument equipped with a 661 nm laser (Wyatt). The whole system is linked to an HPLC system with UV absorbance detection at 280 nm (Agilent) and an Optilab (Wyatt) for differential refractive index (dRI) measurements. Approximately 100 μg of purified hXPR1 protein were injected and flowed through a Superose 6 10/300 GL column (Cytiva) equilibrated with 20 mM Tris pH 7.4, 150 mM NaCl, 0.005% (w/v) GDN, 0.005% (w/v) LMNG, and 0.0001% CHS. Data was analyzed using the Astra software (Wyatt). A d*n*/d*c* of 0.185 is used for the detergent mixture and ε is set to 1.64 ml/mg.cm.

### Cryo-EM sample preparation and data collection

hXPR1 samples in different conditions were concentrated to 10 to 20 mg/mL for cryo-EM grid preparation. Cryo grids were prepared using the Thermo Fisher Vitrobot Mark IV maintained at 8 °C and 100% relative humidity. Quantifoil R1.2/1.3 Cu 300 mesh grids were glow-discharged in air for 15 s using Pelco Easyglow. 3.5 μL hXPR1 sample was applied to each glow-discharged grid. After blotting with filter paper (Ted Pella, Prod. 47000–100) for 3.5– 4.5 s, the grids were plunged into liquid ethane cooled with liquid nitrogen.

Cryo-EM data were collected using Thermo Fisher Titan Krios microscope at 300 kV with a Quantum energy filter (Gatan) with 15eV slit width, and a K3 Summit direct electron detector (Gatan). Movie stacks were collected in super-resolution mode with defocus values ranging between −2.2 μm and −0.8 μm at 105,000x nominal magnification (calibrated per pixel size of 0.416 Å in super-resolution). The exposure time for each stack was 2.6 seconds, fractionated into 40 frames, with a total accumulated dose of 50e^−^/ Å^2^. A total of 16,297 movies were collected for apo-hXPR1 dataset, and 15,802 movies for Pi/InsP_6_-hXPR1 dataset.

### Cryo-EM data processing

For apo-hXPR1, the movie stacks were motion-corrected with MotionCor2^49^ and the aligned final images were binned (2 × 2) to 0.832 Å per pixel size. Dose weighting was performed during motion correction, and the defocus values were estimated with CTFFIND4^50^. After manual curation, a total of 14,168 micrographs were selected which had a CTF-fitted resolution value below 4 Å. A total of 8,468,502 particles were automatically picked using templates from preliminary analysis and extracted for 2D classifications in cryoSPARC^51^. 813,777 particles were selected from the good 2D classes for *ab initio* 3D reconstruction and imported into Relion4.0 for 3D classification^52^. Two good classes with recognizable structural features containing 230,861 particles were selected and imported back to cryoSPARC for non-uniform refinement using C1 symmetry with CTF refinement^53^, which yielded a map with an overall resolution of 3.4 Å. Resolutions were estimated using the gold-standard Fourier shell correlation with a 0.143 cut-off. Local resolution was estimated using ResMap^54^.

The data processing for Pi/InsP6-hXPR1 followed a similar workflow. A total of 14,603 micrographs were selected which had a CTF-fitted resolution value below 4 Å after motion correction and CTF estimation. 11,247,130 particles were automatically picked, with 2,428,881 particles selected from the good 2D classes. A final set containing 536,955 particles were selected after 3D classifications and used for non-uniform refinement using C2 symmetry with CTF refinement, which yielded a map with an overall resolution of 2.3 Å.

### Model building and refinement

The transmembrane domain of apo-hXPR1 was built using the AlphaFold prediction^38^ as the initial model. Carbon backbones and the side chains were adjusted based on the density map. The SPX domain of apo-hXPR1 was built using solved crystal structure^26^ (PDB: 5IJH) as template to perform rigid body docking into the density maps and modified with flexible fitting. The model of Pi/InsP6-hXPR1 was built using the apo-hXRP1 as the initial reference and adjusted based on the density map. Model building was conducted in Coot^55^. Structural refinements were carried out in PHENIX^56^ in real space with secondary structure and geometry restraints. The channel was calculated using CAVER 3.0.3^35^ with a minimum probe radius of 1.2, shell depth of 3, shell radius of 2, and clustering threshold of 3.5.

### Proteoliposome preparation

For proteoliposomes used in Pi transport assay, brain polar lipid extract (Avanti) was mixed with 3% (w/w) cholesterol (Avanti) in chloroform, dried under argon gas stream and further dried overnight in vacuum. Lipids were then hydrated at 10 mg/mL with assay buffer containing 140 mM N-Methyl-D-glucamine (NMDG, Sigma Aldrich), 20 mM HEPES, 1mM phosphoric acid, 10 mM hydrochloric acid, adjusted to pH 7.4 with citric acid. The lipids were flash-frozen in liquid nitrogen, thawed for a total of five freeze-thaw cycles, and then extruded 21 times using polycarbonate filters with a pore size of 50 nm (Whatman) to obtain unilamellar vesicles. 0.01% of DDM was added to destabilize the lipid and then purified wildtype or mutant hXPR1 proteins in 0.03% DDM were added with 1:500 (w/w) protein-to-lipid ratio. The mixture was incubated for 1 hour and detergent was removed by the addition of BioBeads SM-2 (Bio-Rad). Collected liposomes were flash frozen and stored at −80 °C until further use.

Giant unilamellar vesicles (GUVs) used in patch clamp were made from 20 μL brain polar lipid extract with 10% (w/w) cholesterol in chloroform at 5 mg/mL by electroformation using the Vesicle Prep Pro (Nanion Technologies) in 250μL buffer containing 2 mM HEPES at pH 7.4, 1 mM EGTA, 400 mM sorbitol. Purified hXPR1 protein in 0.03% DDM at 0.1 mg/mL was mixed with GUV solution and diluted to a final concentration of approximately 50 to 500 ng/mL (~1:90,000 to ~1:900,000 protein-to-lipid molar ratio) and incubated overnight at 4°C with SM-2 Bio-Beads (Bio-Rad).

### Electrophysiology

Ionic currents were recorded using the patch clamp technique in the inside-out or whole cell configuration. Data were acquired and analyzed as previously described^57^. Traces shown in figures are digitally filtered at 5 kHz. Voltages have been corrected for liquid junction potentials, calculated according to the stationary Nernst–Planck equation using LJPcalc^57^. The bath was grounded through an agar bridge. All experiments were performed at room temperature (22°C–24°C). External solutions contained in mM: **NMDG-MSA** −140 N-methyl-D-glucamine (NMDG), 20 mM HEPES, 5 mM EGTA, 10 HCl, with or without 1 Pi added as phosphoric acid and adjusted to pH 7.2 with 112 methanesulfonic acid (MSA). **NMDG-citrate** −140 NMDG, 20 HEPES, 1 phosphoric acid, 10 HCl, adjusted to pH 7.2 with 17.4 citric acid. **NMDG-Cl** - 10 NMDG, 20 HEPES, 260 Sucrose, pH 7.2 with 8.72 HCl. Internal solutions contained in mM: **20 Pi, 0.1 Ca, K-MSA** - 110 mM KOH, 10 mM K_2_HPO_4_, 10 mM KH_2_PO_4_, 10 HCl, 5 mM HEDTA, with free Ca^2+^ adjusted to 100 μM with CaCl_2_, and pH to 7.2 with 92.5 MSA. **0 Pi, 10 Cl, K-MSA** - 140 mM KOH, 20 mM HEPES, 5 mM EGTA, 10 mM HCl, pH 7.2 with 115.1 MSA. **75 Pi, 10 Cl, K-MSA** - 54 mM K2HPO4, 21 mM KH2PO4, 20 mM HEPES, 5 mM EGTA, 10 mM KCl, pH 7.2 with 16 KOH. **10 Pi, 0 Ca, K-MSA** - as a 13:2 mixture of 0 and 75 Pi, 10 Cl K-MSA. **75 Pi, 0 Cl, K-MSA** - 54 mM K2HPO4, 21 mM KH2PO4, 20 mM HEPES, 5 mM EGTA, pH 7.2 with 16 KOH.

### Proteoliposomal Pi uptake assay

Pi uptake activity was measured with reconstituted proteoliposomes containing either wildtype or mutant hXPR1. The control was empty liposomes. To generate potassium gradient used to perturb the membrane potential, thawed liposomes in assay buffer containing 140 mM NMDG, 20 mM HEPES, 1mM phosphoric acid, 10 mM hydrochloric acid adjusted to pH 7.2 with citric acid were added with 60 mM NaCl and 5 mM KCl. The mixture underwent additional five freeze-thaw cycles using liquid nitrogen. The liposomes were extruded again using 200nm filter membrane for homogeneity, yielding sealed XPR1-containing liposomes with 5 mM internal KCl. The extruded liposomes were exchanged into assay buffer containing 60 mM KCl and 5 mM NaCl using PD-10 desalting column (Cytiva). A total volume of 5 μl of liposomes were added to a 50-μl reaction solution. Carrier non-radioactive sodium phosphate (1M stock, pH 7.4) was added at a final concentration of 25 mM, along with 0.1mCi/mL [^32^P] orthophosphate (5 μCi total, diluted from stock of 8500–9120 Ci/mmole; carrier-free, PerkinElmer) to initiate the reaction. For experiments in which the membrane potential was perturbed, 200 nM valinomycin was added to the reaction mixture. The mixture was incubated for various time points at 37°C. The reaction was rapidly filtered with a G-25 spin column (Cytiva) to remove unincorporated Pi. Radioactivity was determined by liquid scintillation counting.

## Supplementary Material

1

## Data Availability

The atomic coordinates and cryo-EM density maps for hXPR1 in apo state (ligand-free) and in presence of inorganic phosphate and phytic acid have been deposited in the Protein Data Bank (http://www.rcsb.org) with the accession codes 9CKZ and 9CL0, and EMDB (https://www.ebi.ac.uk/pdbe/emdb/) with the codes EMD- 45656 and EMD- 45657, respectively. All electrophysiological data needed to evaluate the conclusions in the paper are present in the paper.
