# Supplementary material for: The identification of XPR1 as a voltage- and phosphate-activated phosphate-permeable ion channel
Source: Res Sq. 2024 Dec 11:rs.3.rs-4457423. Preprint. [Version 1] doi: 10.21203/rs.3.rs-4457423/v1 (PMC11661312; doi:10.21203/rs.3.rs-4457423/v1)
Supplement: 1 [file NIHPPRS4457423V1-supplement-1.pdf]

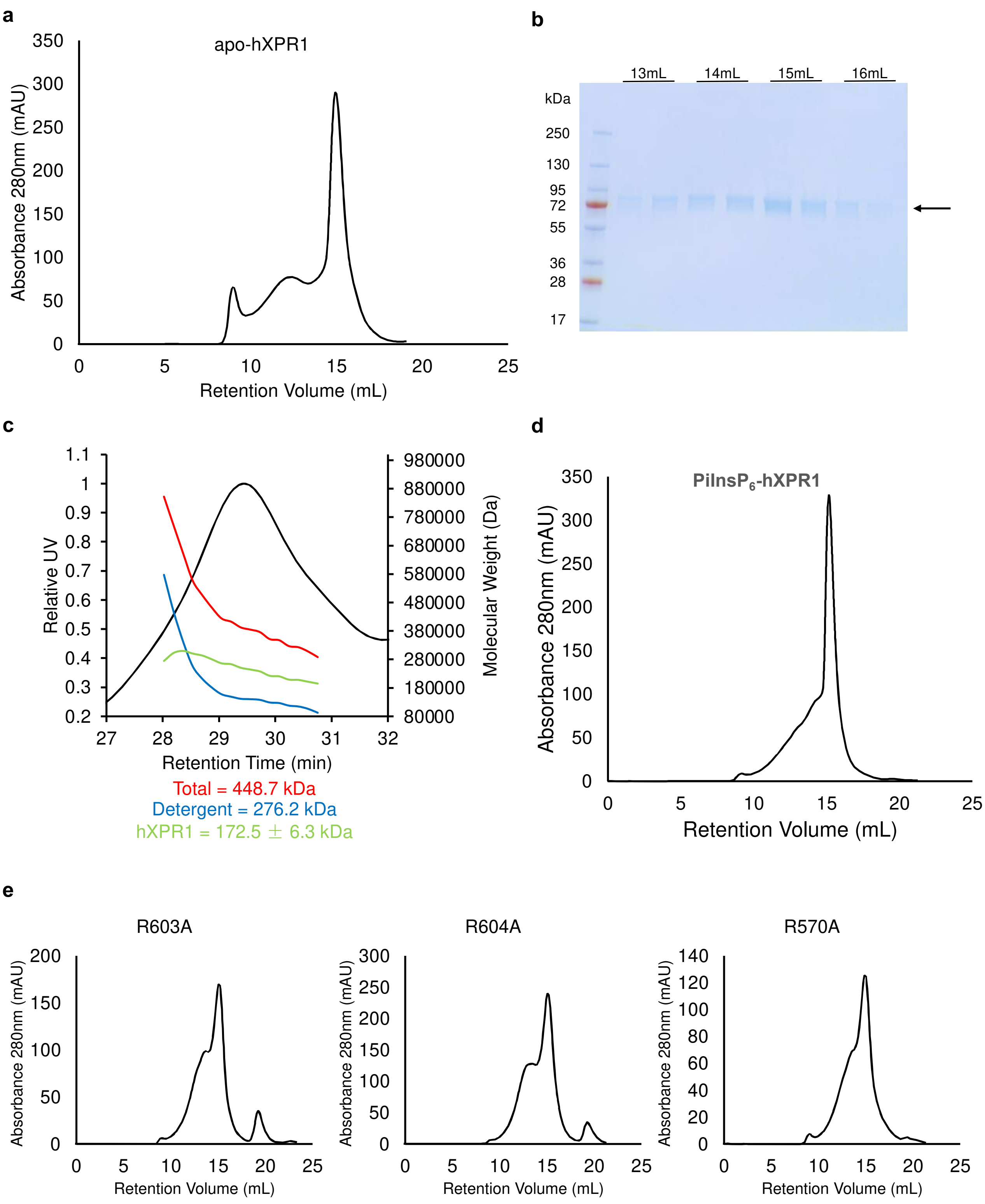

Supplementary Fig. 1: Purification and biochemical characterization of hXPR1.

a. Size-exclusion chromatography (SEC) profile of wildtype hXPR1 in apo-state. **b.** SDS-PAGE profile of the peak fractions from SEC. The arrow indicates the bands corresponding to the purified hXPR1 protein. **c.** SEC-MALS analysis of purified apo-hXPR1 peak fraction. The UV absorption trace for is shown as a black line. The molar masses of the protein–detergent complex (Total, red), the detergent micelle (Detergent, blue) and the protein (hXPR1, green) are indicated. The molecular weight of recombinant hXPR1 monomer is 86.1 kDa. **d.** SEC profile of wildtype hXPR1 in presence of inorganic phosphate and $InsP_6$. **e.** SEC profiles of hXPR1 mutants.

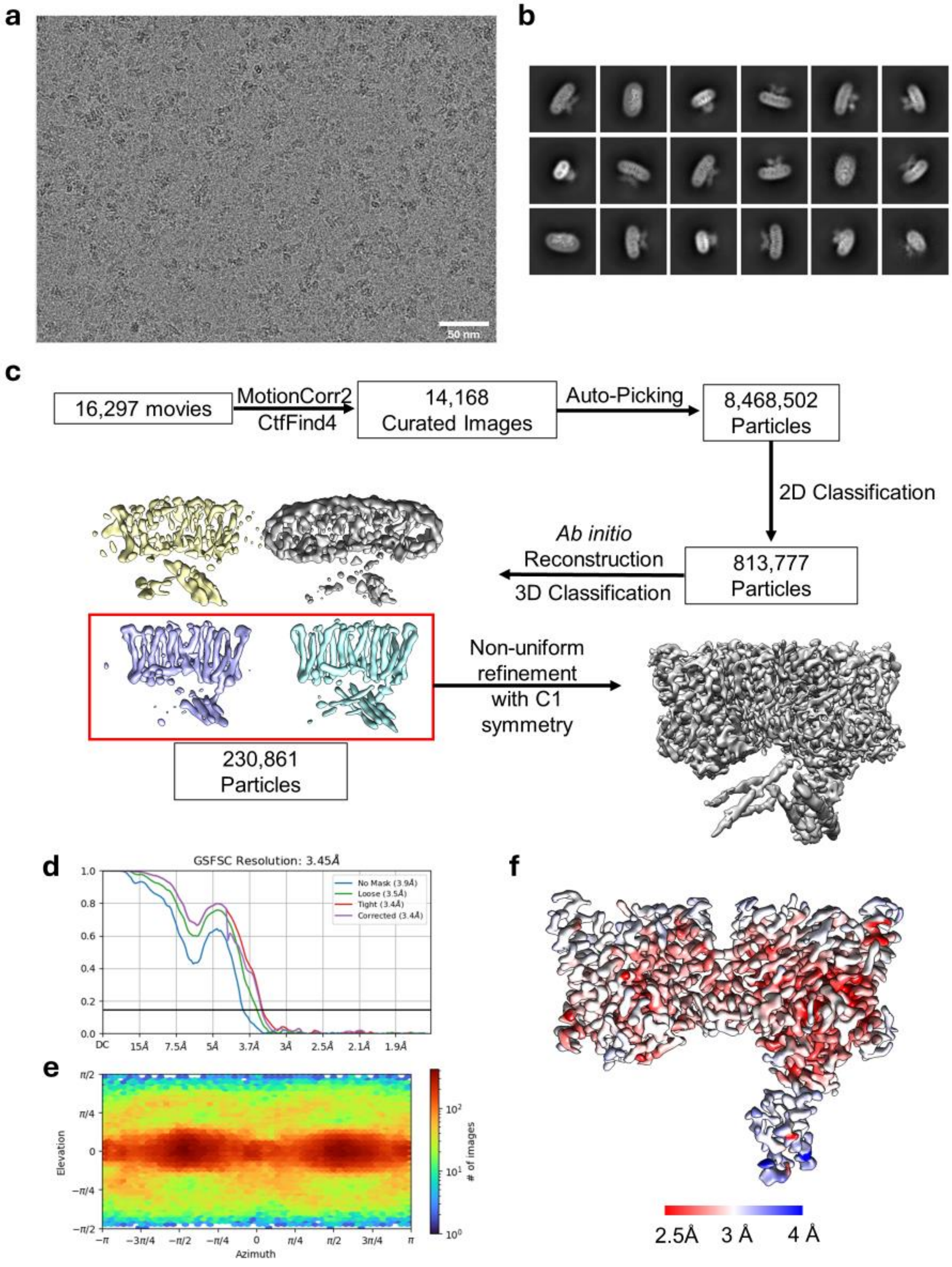

Supplementary Fig. 2: Cryo-EM SPA data processing workflow and the three-dimensional reconstruction map of apo-hXPR1.

a. A representative micrograph of apo-hXPR1 **b.** Representative 2D class averages of apo-hXPR1. **c.** General cryo-EM SPA data processing workflow for apo-hXPR1. **d.** Gold-standard Fourier shell correlation (FSC) curve for the final map of apo-hXPR1. **e.** Angular distribution of particles used in the final reconstruction. **f.** Local-resolution map of apo-hXPR1.

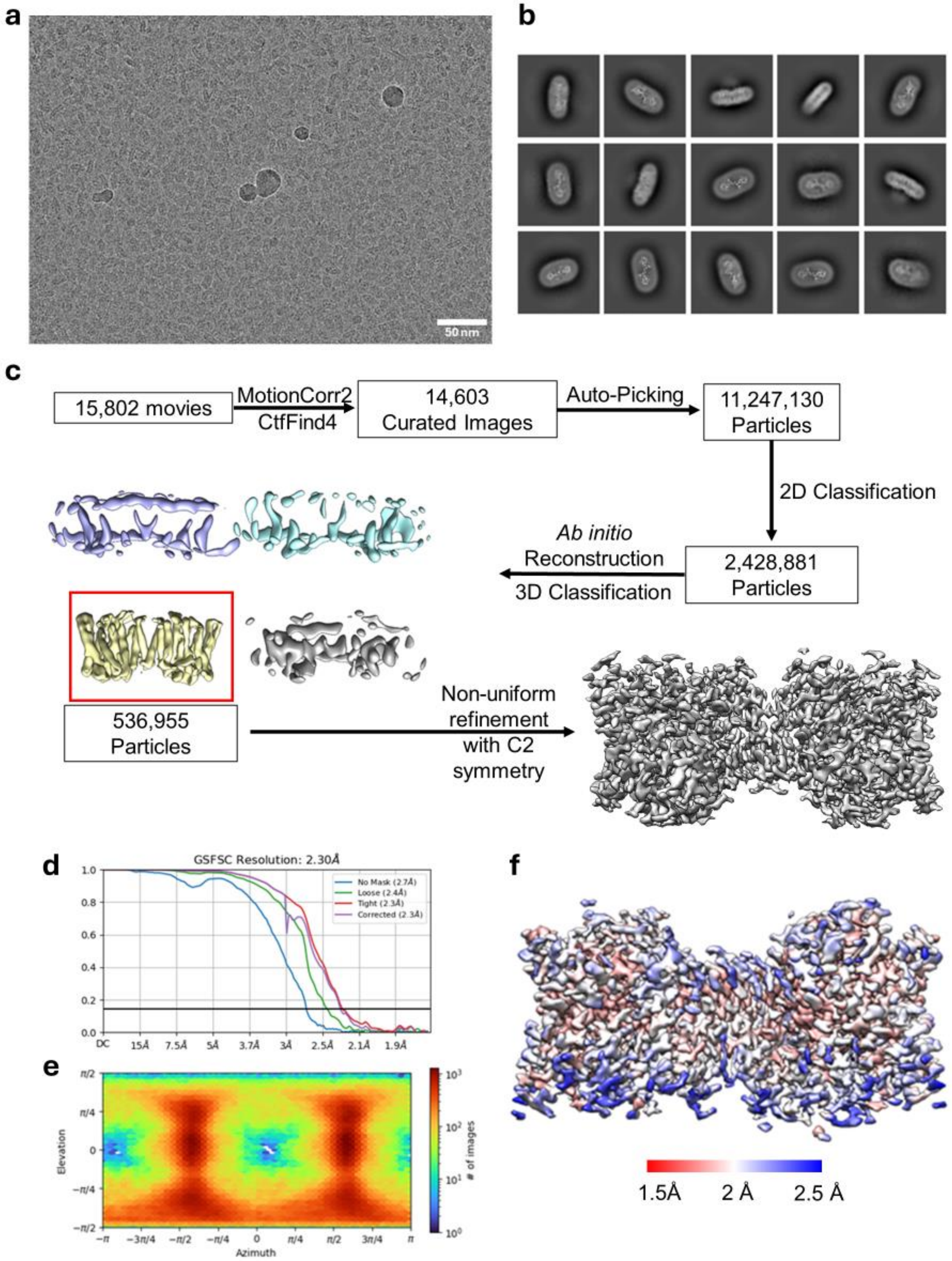

Supplementary Fig. 3: Cryo-EM SPA data processing workflow and the three-dimensional reconstruction map of Pi/InsP$_6$-hXPR1.

a. A representative micrograph of Pi/InsP6-hXPR1 **b.** Representative 2D class averages of Pi/InsP6-hXPR1. **c.** General cryo-EM SPA data processing workflow for Pi/InsP6-hXPR1. **d.** Gold-standard Fourier shell correlation (FSC) curve for the final map of Pi/InsP6-hXPR1. **e.** Angular distribution of particles used in the final reconstruction. **f.** Local-resolution map of Pi/InsP6-hXPR1.

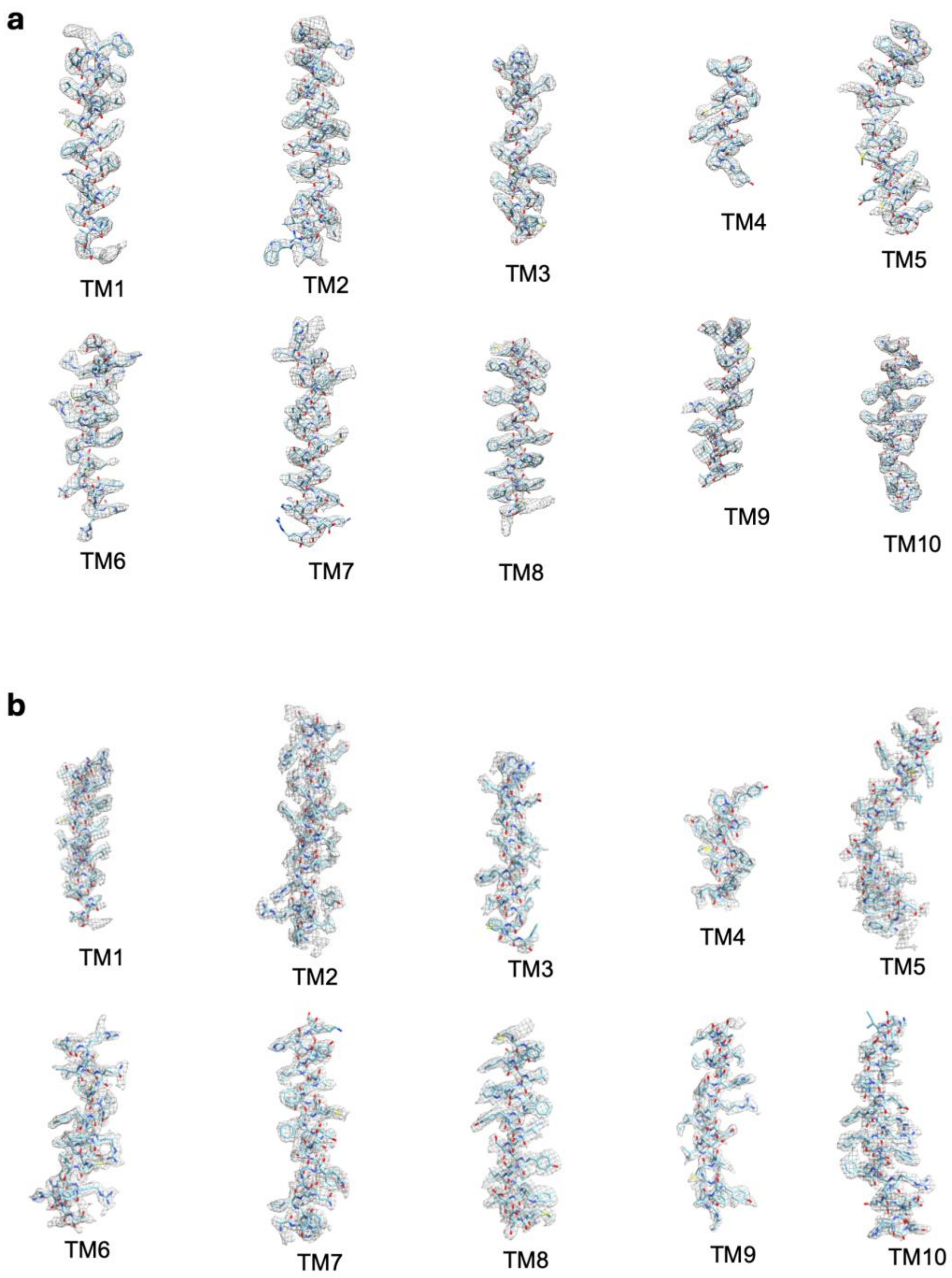

Supplementary Fig. 4: EM densities of the transmembrane helices of hXPR1.
a,b EM density segments (grey mesh) superimposed on the atomic models in stick representation of each transmembrane helix for **a,** apo-hXPR1, and **b,** Pi/$InsP_6$-hXPR1.

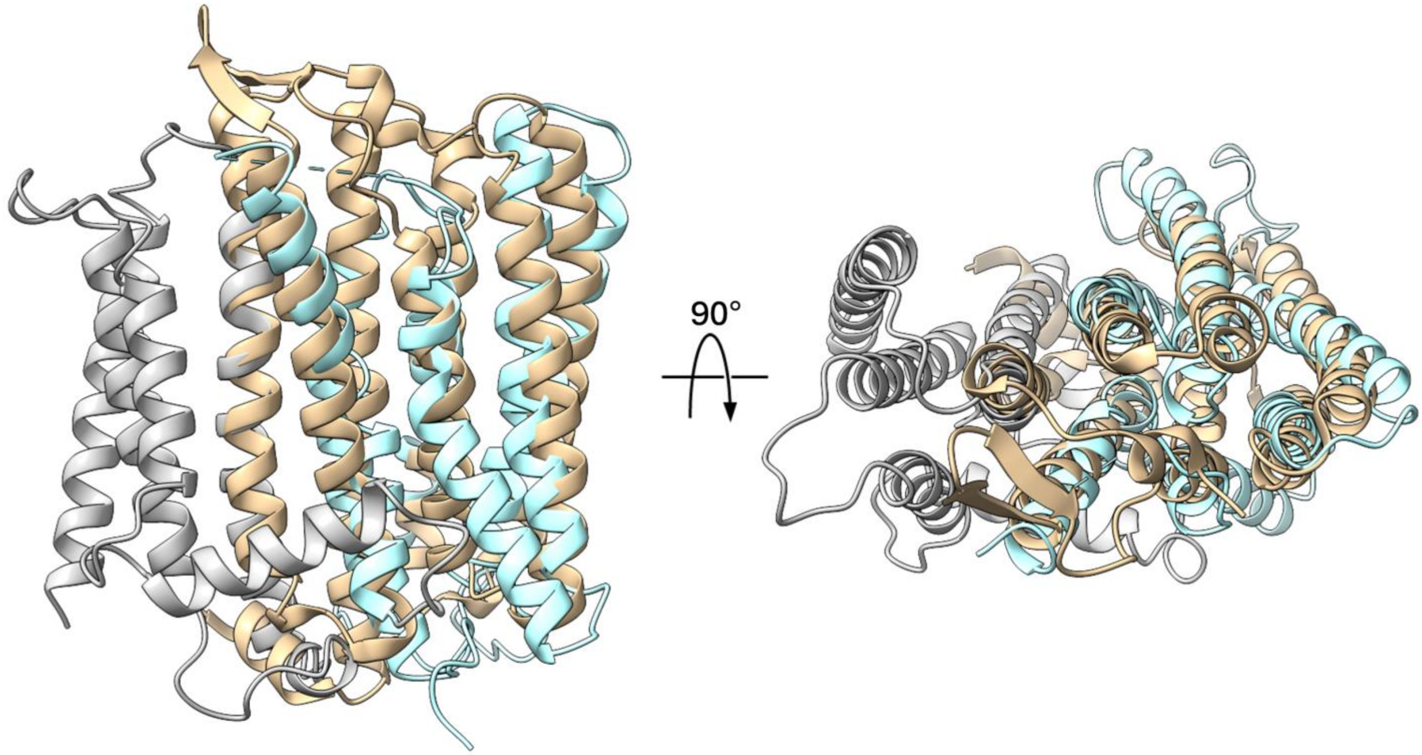

Supplementary Fig. 5: Comparison of apo-hXPR1 TMD to light-driven chloride ion-pumping rhodopsin. The structural comparison based on the DALI similarity search result between the TMD of apo-hXPR1 with EXS domain colored in light cyan and the rest in gray, and its closest resemblance, the light-driven chloride ion-pumping rhodopsin colored in gold (PDB: 5B2N)[34].

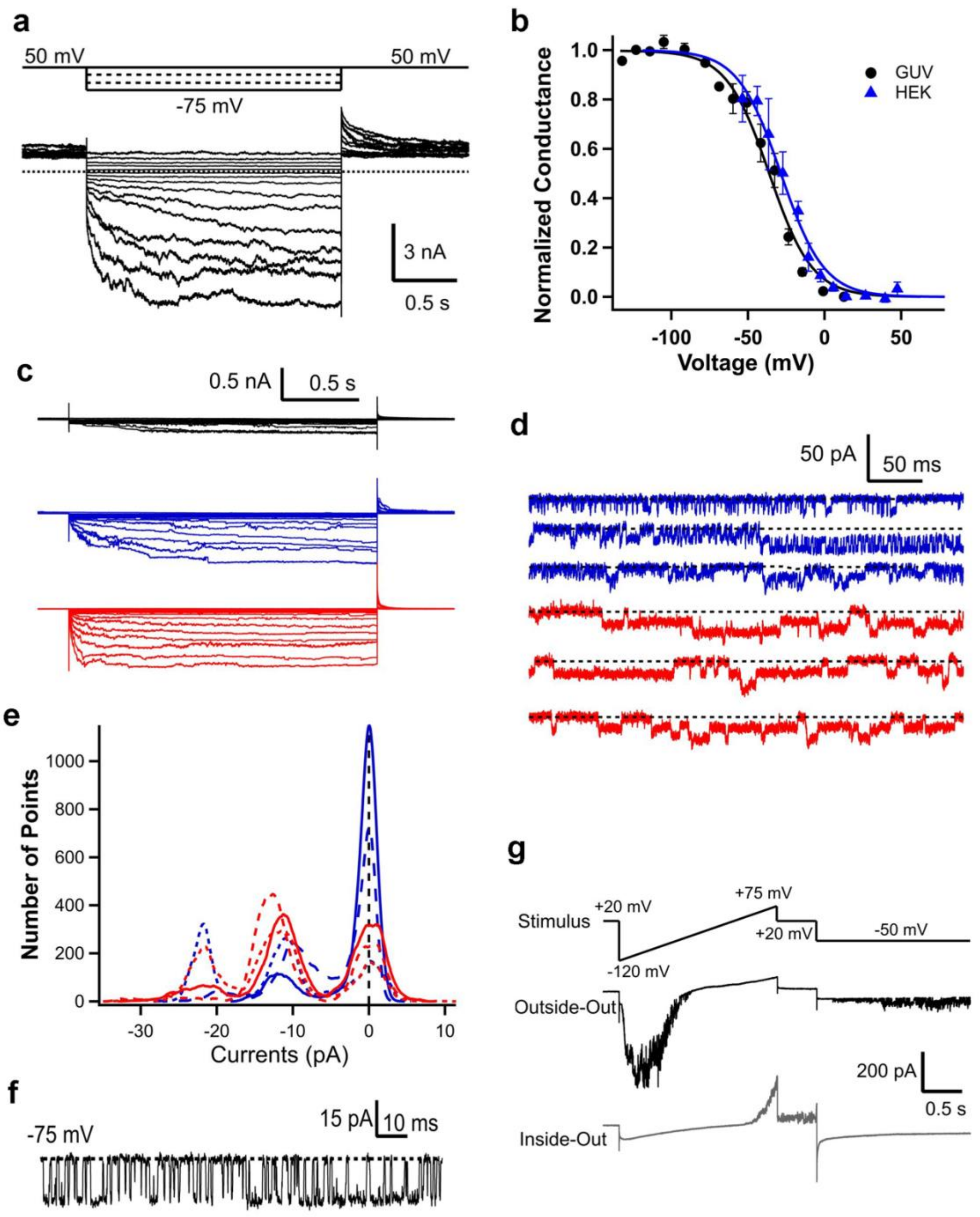

Supplementary Fig. 6: Additional electrophysiological recordings of hXPR1.
a. Whole cell XPR1 currents from HEK293S cells evoked by pulses to different voltages. **b.** G-V relation measured from tail currents in a. are similar to the mean G-V for XPR1 measured from GUV under the same solution conditions (both fit with Boltzman function with z = -1.8 *e*). **c.** XPR1 currents evoked at different voltages (+40 to –100 mV) from a GUV are increased and activate more rapidly as internal [Pi] is increased

from 0 (black) to 10 mM (blue) to 75 mM (red) using 10 Cl K-MSA internal solutions with external 0 Pi NMDG-MSA. **d.** Selected traces showing unitary current fluctuations in macroscopic currents at –50 mV with 10 mM (blue) or 75 mM (red) internal Pi from the same patch as c. **e.** All points amplitude histograms for traces in **d.** indicate single channel current amplitude is similar with different [Pi]. **f.** Steady state unitary current fluctuations in 0 Pi at –75 mV**. g.** Currents recorded from excised GUV patches when the external side was exposed to 20 mM Pi, 0.1 Ca K-MSA solution and internal side to NMDG-MSA solution demonstrate that XPR1 oriented with extracellular side facing the lumen can be selectively activated by external Pi. Lower panel shows outward currents evoked at positive voltages in inside-out recording. Upper panel recorded in the same patch in outside-out mode reproduces the current rectification observed in inside-out mode with high luminal Pi (Fig. 2a).

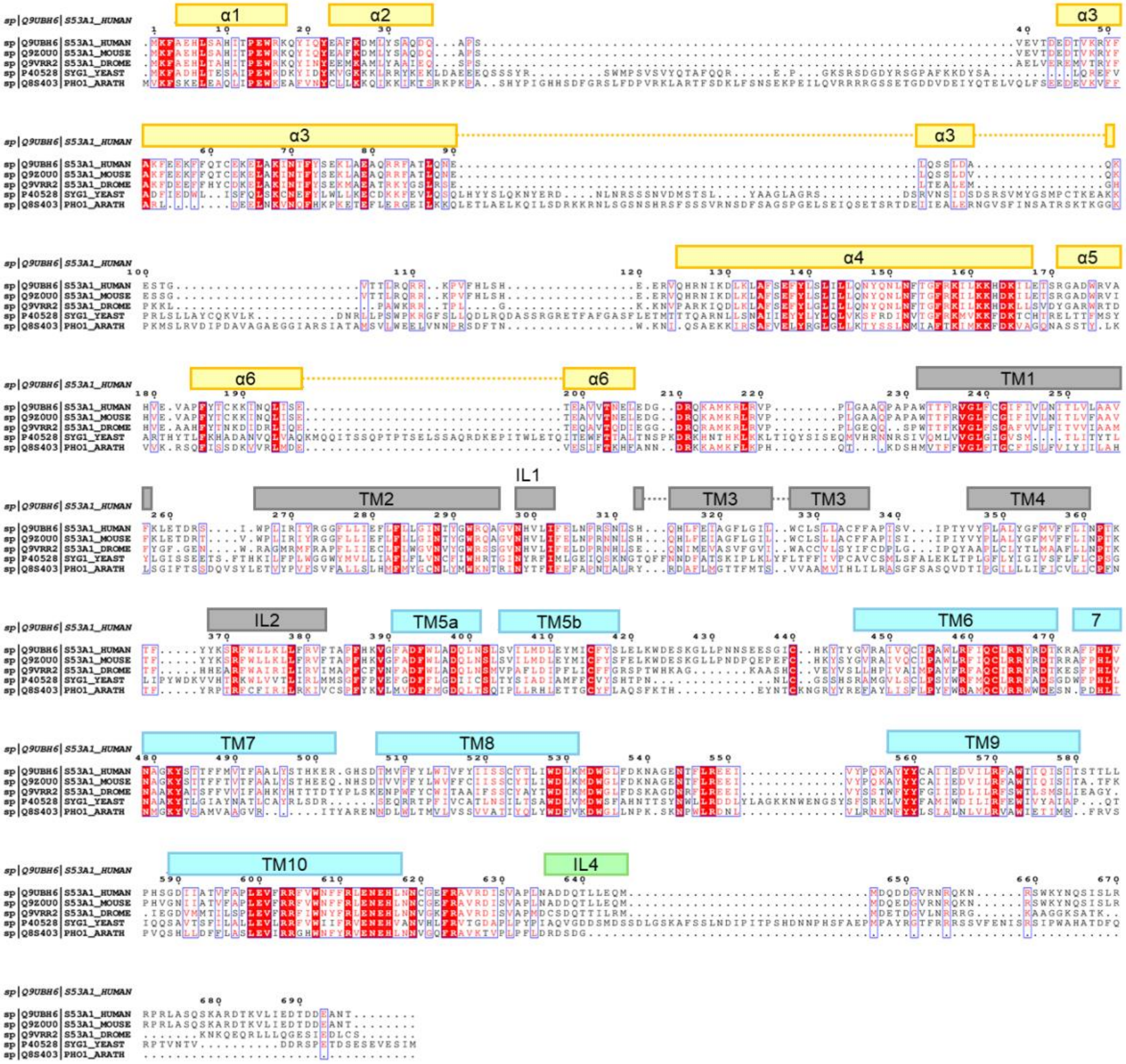

Supplementary Fig. 7: Amino acid sequence alignment of XPR1 homologues.

Amino acid sequences of human XPR1 (Uniprot: Q9UBH6), mouse XPR1 (Uniprot: Q9Z0U0), *D. melanogaster* PXo (Uniprot: Q9VRR2), *S. cerevisiae* SYG1 (Uniprot: P40528), and *A. thaliana* PHO1 (Uniprot: Q8S403) are aligned using the Clustal Omega Server. Residues with red background are conserved, and ones in blue boxes show similar residues with partial conservation between homologs in red letters. Secondary structures are shown on the top of the sequences with SPX domain colored in yellow, TM5-10 in blue, IL4 in green, and the rest in grey.

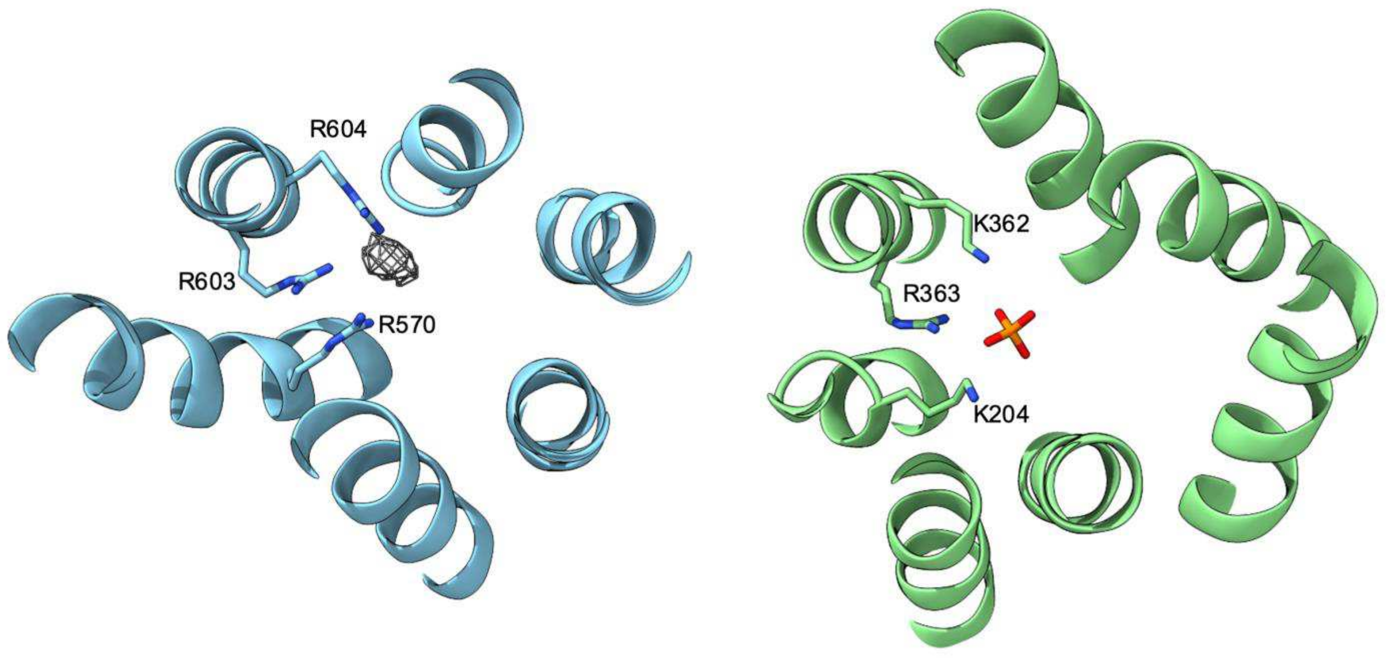

Supplementary Fig. 8: Comparison of the putative Pi coordination site in hXPR1 to the Pi binding site in GsGPT.

The putative Pi coordination site in hXPR1 with three key arginine residues shown in stick model with the potentially coordinated ion density in gray mesh in the map of Pi/InsP$_6$-hXPR1(left), and the Pi-binding site in GsGPT (PDB:5Y78)[36] with three key positive residues and the bound phosphate ion shown in stick model (right).

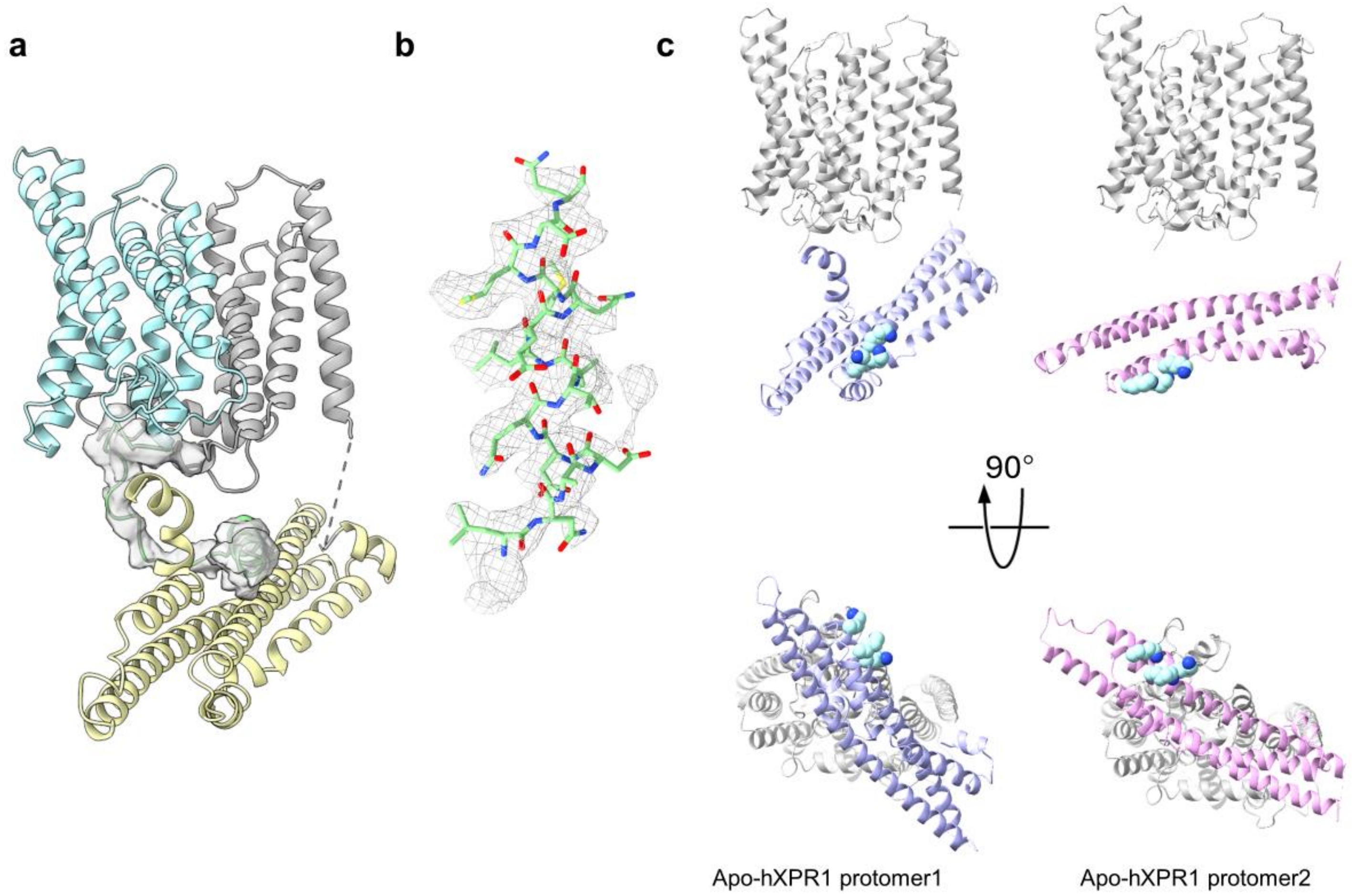

Supplementary Fig. 9: The C-terminal cytoplasmic tail connects TMD to SPX domain.
a. The structure of one apo-hXPR1 protomer with the cytosolic C-terminal tail colored in green, fitted in the low-pass filtered map of the tail (grey surface). **b.** The secondary structure of IL4 helix fitted with the EM density (grey mesh). **c.** The relative orientations of cytosolic domain with respect to the invariant TMD between two protomers in apo-hXPR1 viewed in the membrane plane (top) and from cytoplasm (bottom). The lysine surface cluster residues[26] are shown in space-filling model colored in light cyan.

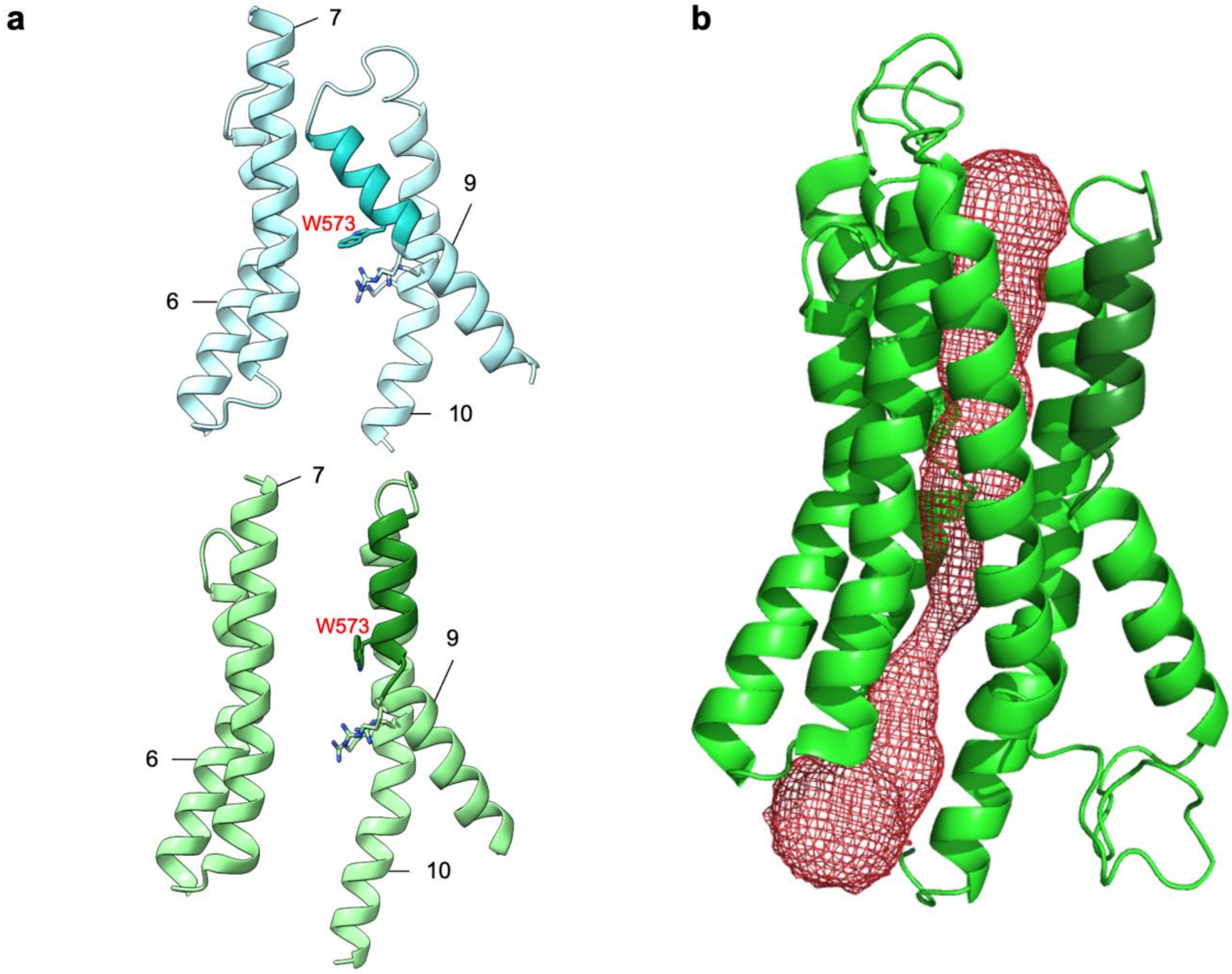

Supplementary Fig. 10: Comparison to the AlphaFold2 prediction.

a. The structure of the putative pore with core residues Arg570, Arg603, Arg604 and Trp573 shown in stick representation for apo-hXPR1 with the extracellular segment of TM9 colored in dark cyan (top, light cyan), and for AlphaFold2 prediction with the extracellular segment of TM9 colored in dark green (bottom, light green). **b.** The continuous tunnel (red mesh) from cytoplasm to extracellular space identified within TM5-10 of AlphaFold2 prediction using CAVER3.

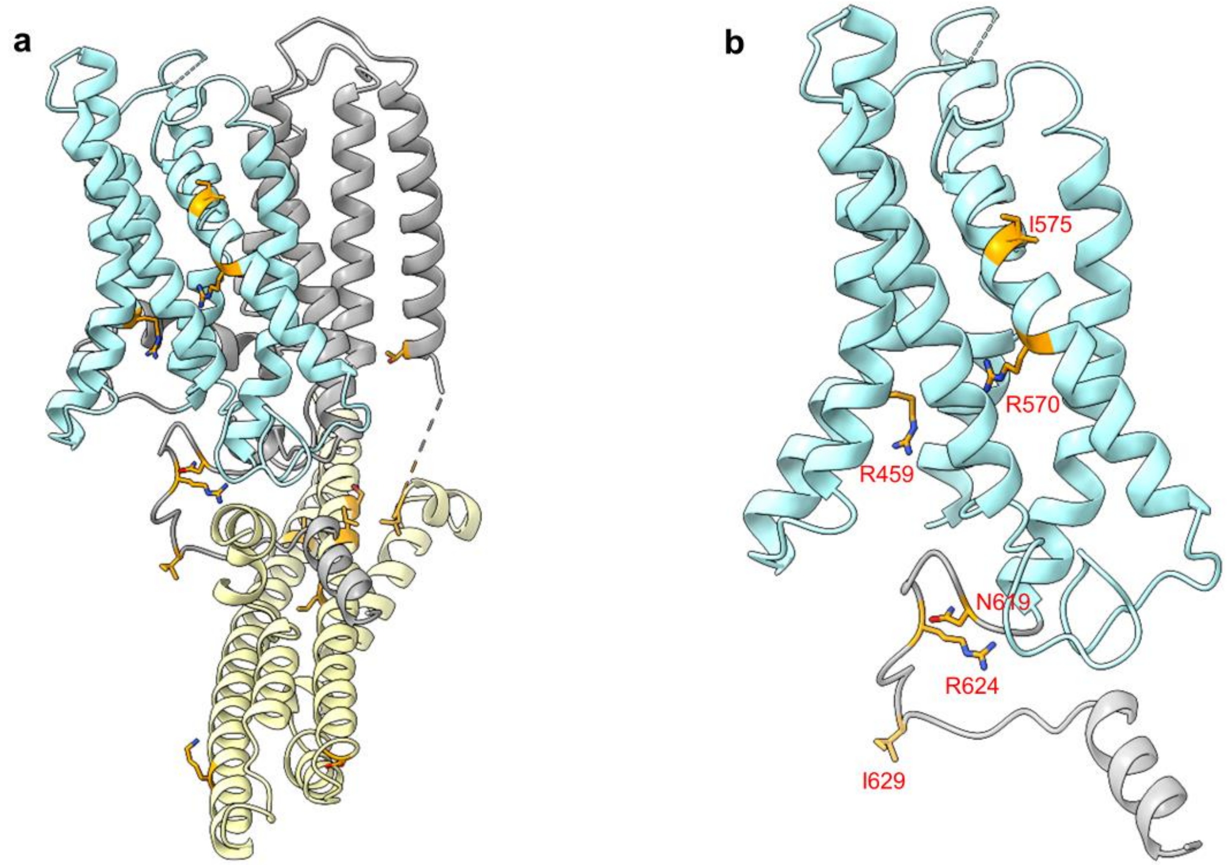

Supplementary Fig. 11: Locations of PFBC mutations.

a. Structure of hXPR1 with PFBC mutants shown in the gold-colored stick representation. **b.** Close-up views on the mutants located in the putative pore and the cytoplasmic C-terminal tail.

	Apo-hXPR1 (PDB: 9CKZ) (EMDB: 45656)	Pi/InsP6-hXPR1 (PDB: 9CL0) (EMDB: 45657)
**Data collection and processing**		
Instrument	Titan Krios (Thermo Fisher)	Titan Krios (Thermo Fisher)
Detector	K3 Summit (Gatan)	K3 Summit (Gatan)
Magnification	105,000x	105,000x
Voltage (kV)	300	300
Total electron dose (e^-/$Å^2$)	50	50
Defocus Range (µm)	-0.8 to -2.2	-0.8 to -2.2
Pixel size ($Å^2$)	0.832	0.832
Symmetry imposed	C1	C2
Micrograph collected (N)	16,297	15,802
Initial particle images(N)	8,468,502	11,247,130
Final particle images(N)	230,861	536,955
Map resolution (Å)	3.45	2.30
FSC threshold	0.143	0.143
Map sharpening B-factors($Å^2$)	-125.5	-88.2
**Refinement**		
Initial model used	AlphaFold2 Prediction (Uniprot: Q9UBH6)	apo-hXPR1
Model resolution(Å)	3.5	2.4
FSC threshold	0.5	0.5
**Validation**		
B factors ($Å^2$) (mean)	66.16	4.30
Bond lengths (Å)	0.005	0.006
Bond angles (°)	0.810	1.621
MolProbity score	1.78	2.23
Clash score	8.46	11.26
**Ramachandran plot**		
Favored (%)	95.35	94.61
Allowed (%)	4.20	5.12
Disallowed (%)	0.45	0.27

Supplementary Table 1: Summary of cryo-EM data collection, processing, and structural refinement.
